# OVERCOMING CHALLENGES TO PAIN ASSESSMENT AND FACTORS THAT INFLUENCE PAIN IN PATIENTS WITH DEMENTIA

**DOI:** 10.1093/geroni/igac059.1534

**Published:** 2022-12-20

**Authors:** Ashley Kuzmik

**Affiliations:** Penn State University, University Park, Pennsylvania, United States

## Abstract

The purpose of this study was to describe the optimal way to measure pain among older hospitalized patients with dementia and evaluate the factors that influence pain. The PAINAD is described as a reliable and valid observation measure of pain in this population based on Rasch analysis and was invariant to gender or racial biases. Using this measure and a protocol for observation of pain, pain and associated factors were obtained on the first 112 participants from 6 hospitals in the FFC-AC-EIT study. For descriptive purposes and to guide interventions, factors that were associated with pain were tested. The following factors explained 61% of the variance: functional focused care activities performed, delirium, and quality of care interactions. Those that performed more functional activities, had less delirium, and had higher (more positive) quality of care interactions were less likely to have pain. The findings also supported the use of the PAINAD.

